# The effects of metformin in type 1 diabetes mellitus

**DOI:** 10.1186/s12902-017-0228-9

**Published:** 2018-01-16

**Authors:** Selvihan Beysel, Ilknur Ozturk Unsal, Muhammed Kizilgul, Mustafa Caliskan, Bekir Ucan, Erman Cakal

**Affiliations:** 1 Department of Endocrinology and Metabolism, Eskisehir State Hospital, Eskisehir, Turkey; 2 Department of Medical Biology, Baskent University, Ankara, Turkey; 3 Department of Endocrinology and Metabolism, Ankara Diskapi Teaching and Research Hospital, Ankara, Turkey; 4 Department of Endocrinology and Metabolism, Kilis State Hospital, Kilis, Turkey; 5 Department of Endocrinology and Metabolism, Duzce Ataturk State Hospital, Duzce, Turkey

**Keywords:** Metformin, Type 1 diabetes, Insulin requirement

## Abstract

**Background:**

This retrospective study investigated the effect of adding metformin to pharmacologic insulin dosing in type 1 diabetics on insulin therapy 1 year after treatment compared with patients on insulin therapy alone.

**Methods:**

Twenty-nine adults with type 1 diabetes who had metformin added to their insulin therapy for 12 months were compared with 29 adults with type 1 diabetes who remained on insulin-alone therapy.

**Results:**

Fifty-eight patients with C peptide negative-type 1 diabetics (26 females, mean age: 29.01 ± 7.03 years, BMI: 24.18 ± 3.16 kg/m2) were analyzed. Age, sex, body weight, insulin dose requirement, plasma glucose (PG), blood pressure (BP), and lipids did not differ between groups before treatment (*p* > 0.05). Metabolic syndrome (44.8 vs 41.4%, *p* > 0.05) did not differ between the metformin-insulin and insulin alone groups before treatment. Metabolic syndrome was more decreased in the metformin-insulin group than in the insulin alone group after treatment (−8.9 ± 1.3 vs. 2.5 ± 0.6%, *p* = 0.028). Insulin dose requirement was lower in the metformin-insulin group than in the insulin alone group (−0.03 vs. 0.11 IU/kg/d, *p* = 0.006). Fasting PG (−26.9 ± 54.2 vs. 0.7 ± 29.5 mg/dL, *p* = 0.022) and postprandial PG (−43.1 ± 61.8 mg/dL vs. −3.1 ± 40.1 mg/dL, *p* = 0.010) was more decreased in the metformin-insulin group than in the insulin alone group. Body weight, lipids, and HbA1c did not differ between the groups (*p* > 0.05).

**Conclusions:**

Metformin decreased glucose concentrations, reduced metabolic syndrome, as well as insulin dose requirement more than insulin therapy alone, 1 year after treatment. These results were independent of blood lipid improvement or weight loss, although on average weight remained decreased with metformin-insulin therapy, whereas the average weight increased with insulin therapy alone.

## Background

Despite intensive insulin therapy, target hemoglobin A_1c_ (HbA_1c_) levels remain above 7.0% in many patients with type 1 diabetes mellitus (DM) with poor metabolic control [1]. Standard insulin therapy in type 1 diabetes has been associated with increased complications including hypoglycemia, weight gain, and dyslipidemia [2]. Insulin-stimulated skeletal muscle glucose uptake as well as insulin action reduces in type 1 diabetics. This effect contributes to the development of insulin resistance [3, 4]. Insulin resistance leads to poor glycemic control and chronic complications in type 1 diabetics [5, 6]. Metabolic syndrome is a clinical proxy for insulin resistance. Type 1 diabetes associated with metabolic syndrome has been termed as double diabetes [7–9]. Obesity [10], lack of exercise [11], and puberty [12] are primary causes of insulin resistance in type 1 diabetes. Management of insulin resistance usually requires an increase in insulin dose requirement. Increased insulin dose requirement might cause weight gain and hypoglycemia, which might lead to noncompliance with therapy and ultimately poor glycemic control [13].

Metformin is an oral anti-hyperglycemic agent and commonly used in the treatment of type 2 diabetes. It increases both hepatic and peripheral insulin sensitivity in the liver by inhibiting basal hepatic glucose production, as well as in skeletal muscles and adipocytes, by increasing glucose uptake [4, 14, 15]. Thus, it enhances insulin action and improves glycemic control. Metformin leads to reduce insulin dose requirement as well as weight gain because it increases insulin sensitivity. In this respect, compared with insulin monotherapy, the addition of metformin to insulin therapy improves metabolic control and decreases complications in type 2 diabetes [16]. Metformin has been shown to increase insulin sensitivity [17] and reduce metabolic syndrome incidence in people with prediabetes [18]. The addition of metformin to insulin therapy in type 1 DM is still under debate. Until now, a limited number of studies have investigated the addition of metformin to insulin therapy in type 1 diabetics [4, 19–22]. Metformin as an adjunctive therapy is not formally recommended in type 1 diabetes unlike in type 2 diabetes [23].

This retrospective study investigated the effect of adding metformin to pharmacologic insulin dosing in type 1 diabetics on insulin therapy 1 year after treatment compared with patients on insulin therapy alone. This study aimed to investigate the effect of metformin, as an adjunctive therapy, on the treatment of poorly controlled type 1 diabetics.

## Methods

Adults with C-peptide-negative type 1 diabetes were treated in Diskapi Yildirim Beyazit Training and Research Hospital, Endocrinology and Metabolism Department, between January 2010 and February 2013. Twenty-nine patients with type 1 diabetes had metformin added to their insulin therapy for 12 months. These patients were compared with 29 adults with type 1 diabetes who remained on insulin-alone therapy for 12 months. The inclusion criteria were as follows: age between 18 and 60 years, lack of metabolic control (HbA_1c_ above 7.5% despite intensive insulin treatment), and complete medical data records. The exclusion criteria were as follows: lack of treatment adherence, renal impairment (estimated glomerular filtration rate lower than 60 mL/min) and liver disease (aminotransferase level higher than twice the upper normal limit). The patients were examined every 3 months. This retrospective study was approved and an informed consent was obtained from the patients.

### Clinical outcomes

Metabolic syndrome, hypoglycemia, and drug adverse effects were recorded. Insulin dose requirement, blood pressure (BP), HbA1c, body mass index (BMI), fasting plasma glucose (FPG), postprandial plasma glucose (PPG), total cholesterol, triglyceride and low-density lipoprotein-cholesterol (LDL-C) and high-density lipoprotein-cholesterol (HDL-C) were compared before and after treatment. FPG and PPG were measured twice and average value was recorded. Insulin therapy and daily total insulin dose per bodyweight (IU/kg/d) were recorded. Office BP was measured before and after treatment. Office BP was measured with patients in the sitting position after 5 min of rest, provided that the arm was supported at heart level and the BP cuff covered about 80% of the circumference of the upper arm with the lower edge 2.5–3 cm above the elbow. Waist-circumference (WC) was measured midway between the lower costal margin and iliac crest, and hip circumference was measured at the height of the greater trochanter. Metabolic syndrome was defined according to the Adult Treatment Panel III criteria, and its diagnosis required three or more of the following: [1] WC ≥ 94 cm for men and ≥80 cm for women, [2] triglyceride ≥150 mg/dL, [3] HDL-C < 40 mg/dL for men and <50 mg/dL for women, [4] fasting glucose levels ≥100 mg/dL, and [5] systolic BP ≥130 mmHg and diastolic BP ≥ 85 mmHg [24].

### Statistical analysis

Statistical analysis was performed using SPSS 18.0 (SPSS, Inc) software. Descriptive analyses are expressed as mean ± standard deviation (SD), percentages (%), median (min-max), odds ratio (OR), and 95% confidence intervals (CI). Kolmogorov-Smirnov or Shapiro-Wilk W was used for normality. The Chi-square test or Fisher’s exact test, where appropriate, was used for categorical variables. Student’s t-test was used for normally distributed continuous variables. The Mann-Whitney U test was used for nonparametric variables. McNemar’s test was used for categorical variables before and after treatment. The paired samples t-test was used for parametric variables and the Wilcoxon test was used for nonparametric variables before and after treatment. The association between metabolic syndrome and insulin requirement was tested using Spearman’s correlation coefficients. *p* < 0.05 was accepted as statistically significant.

## Results

The patients with type 1 diabetes (26 females, mean age: 29.01 ± 7.03 years, BMI: 24.18 ± 3.16 kg/m^2^) were retrospectively analyzed. The mean duration of diabetes was 12.05 ± 6.53 years. The mean metformin dose was 2124.2 ± 524.2 mg/d. Some 3.4% of patients in the metformin-insulin group were on continuous subcutaneous insulin infusion therapy and 96.6% were under intensified insulin therapy. All patients in insulin alone group were on intensified insulin therapy. There was no significant difference between the groups regarding age, sex, duration of diabetes, BMI, body weight, insulin dose requirement, waist circumference, systolic and diastolic blood pressure, lipids, hypertension, dyslipidemia, overweight/obesity and prevalence, and risk factors of metabolic syndrome before treatment (*p* > 0.05) (Table 1).

**Table 1:** Characteristics of groups before treatment

Variables	Metformin-insulin *n* = 29	İnsulin-only *n* = 29	*p* value
Female (%)	41.4	48.3	0.597
Metabolic syndrome (%)	44.8	41.4	0.585
Overweight/obesity (%)	31.0	24.1	0.291
Hypertension (%)	27.6	13.8	0.195
Hyperlipidemia (%)	31.0	37.9	0.581
Antihypertensive drugs (%)	12.3	7.2	0.127
Antilipidemic drugs (%)	14.1	16.7	0.895
Age (yr)	30.4 ± 7.5	27.5 ± 6.3	0.127
Duration of diabetes (yr)	13.1 ± 6.8	10.9 ± 6.1	0.173
Body weight (kg)	69.23 ± 10.13	66.96 ± 11.50	0.429
BMI (kg/m^2^)	24.43 ± 2.87	24.02 ± 3.46	0.446
Waist circumference (cm)	82.39 ± 8.28	80.65 ± 9.59	0.368
Risk factors of metabolic syndrome (n)	2.27 ± 1.13	2.31 ± 1.03	0.904
Creatinine (mg/dl)	0.81 ± 0.19	0.79 ± 0.14	0.124
FPG (mg/dl)	192.37 ± 56.01	174.48 ± 53.60	0.232
PPG (mg/dl)	277.31 ± 57.43	250.04 ± 64.35	0.100
HbA_1c_ (%)	9.55 ± 1.43	9.22 ± 1.79	0.441
Total cholesterol (mg/dl)	183.43 ± 59.45	177.25 ± 26.89	0.836
Triglycerides (mg/dl)	141.93 ± 131.33	136.96 ± 117.98	0.844
HDL cholesterol (mg/dl)	54.96 ± 13.59	50.28 ± 11.68	0.233
LDL cholesterol (mg/dl)	105.46 ± 39.25	107.52 ± 27.26	0.566
Systolic blood pressure (mmHg)	122.75 ± 16.74	119.48 ± 16.70	0.441
Diastolic blood pressure (mmHg)	78.13 ± 12.76	76.89 ± 11.83	0.690
Total daily insulin dose (IU/kg body weight/d)	0.92 ± 0.27	0.98 ± 0.30	0.232

Data are shown as mean ± standard deviation (mean ± SD) or percentage (%)

*Abbreviations*: *BMI* body mass index, *FPG* fasting plasma glucose, *HbA*_*1c*_ hemoglobin A_1c_, *HDL* high density lipoprotein, *LDL* low density lipoprotein, *PPG* postprandial plasma glucose, *IU* international units

Change in body weight (−0.41 ± 2.44 vs. 0.13 ± 2.55 kg, *p* > 0.05) and waist circumference (−0.34 ± 1.67 vs. 0.41 ± 1.61, *p* > 0.05) did not differ between the metformin-insulin and insulin alone groups after treatment. The increase in systolic BP (6.72 ± 6.16 vs. 1.55 ± 11.02 mmHg, *p* = 0.032) and diastolic BP (4.82 ± 7.84 vs. 0.48 ± 6.41 mmHg, *p* = 0.025) was significantly higher in the insulin alone group than in the metformin-insulin group (Table 2).

**Table 2:** Change in metabolic parameters after treatment

Variables	Metformin-insulin *n* = 29	İnsulin-only *n* = 29	*p* value
∆ Metabolic syndrome prevalence (%)	−8.9 ± 1.3	2.5 ± 0.6	**0.028**
∆ Body weight (kg)	−0.41 ± 2.44	0.13 ± 2.55	0.222
∆ Waist circumference (cm)	−0.34 ± 1.67	0.41 ± 1.61	0.384
∆ FPG (mg/dl)	−26.9 ± 54.2	0.7 ± 29.5	**0.022**
∆ PPG (mg/dl)	−43.1 ± 61.8	−3.1 ± 40.1	**0.010**
∆ HbA_1c_ (%)	−0.8 ± 1.4	−0.3 ± 1.3	0.075
∆ Total cholesterol (mg/dl)	−2.78 ± 36.7	−2.20 ± 17.9	0.962
∆ Triglycerides (mg/dl)	−21.5 ± 98.6	−26.2 ± 75.5	0.855
∆ LDL-C (mg/dl)	−5.0 ± 27.6	−4.9 ± 23.8	0.899
∆ HDL-C (mg/dl)	0.43 ± 9.65	2.88 ± 9.50	0.522
∆ Systolic blood pressure (mmHg)	1.55 ± 11.02	6.72 ± 6.16	**0.032**
∆ Diastolic blood pressure (mmHg)	0.48 ± 6.41	4.82 ± 7.84	**0.025**
∆ Total daily insulin dose (IU/kg bodyweight/d)^a^	−0.03 (−0.39 ─ 0.28)	0.11 (−0.24 ─ 0.35)	**0.006**

Data are shown as mean ± standard deviation (means ± SD) and ^a^median (min-max)

The difference (∆) are shown as change in values before and after treatment (after value minus before value)

*Abbreviations*: *FPG* fasting plasma glucose, *HbA*_*1c*_ hemoglobin A_1c_, *PPG* postprandial plasma glucose, *HDL-C* high density lipoprotein-cholesterol, *LDL-C* low density lipoprotein-cholesterol, *IU* international units

Bold represents the significant *p*-values

Insulin dose requirement decreased by 0.03 IU/kg/d in the metformin-insulin group, whereas it increased by 0.11 IU/kg/d in the insulin alone group after treatment. Insulin dose requirement was significantly increased in the insulin alone group compared with the metformin-insulin group (*p* = 0.006) (Table 2).

Metabolic syndrome prevalence (44.8 vs. 41.4%, *p* > 0.05) did not differ between the metformin-insulin group and the insulin alone group before treatment. Metabolic syndrome prevalence was significantly decreased in the metformin-insulin group compared with the insulin alone group after treatment (−8.9 ± 1.3 vs. 2.5 ± 0.6%, *p* = 0.028, Table 2). The mean risk factors of metabolic syndrome was decreased in the metformin-insulin group (2.27 ± 1.13 vs. 2.03 ± 0.94, *p* = 0.06), whereas it did not change in the insulin alone group (2.31 ± 1.03 vs. 2.20 ± 0.94, *p* = 0.264). The patients in the metformin-insulin and insulin alone groups were divided into subgroups as metabolic syndrome and control non-metabolic syndrome. Metabolic syndrome percentage was decreased in the metformin-insulin group (44.8 vs. 37.9%, *p* = 0.008) whereas it increased in the insulin alone group (41.4 vs. 44.8%, *p* = 0.035).

A decrease in FPG of 26.9 ± 54.2 mg/dL and PPG of 43.1 ± 61.8 mg/dl was observed in the metformin-insulin group after treatment. A decrease in PPG of 3.1 ± 40.1 mg/dL and an increase in FPG of 0.7 ± 29.5 mg/dL were observed in the insulin alone group. There was a significant reduction in FPG (*p* = 0.022) and PPG (*p* = 0.010) in the metformin-insulin group compared with the insulin alone group. The decrease in HbA_1c_ was not significantly different between the groups (−0.8 ± 1.4 vs. −0.3 ± 1.3%, *p* > 0.05). Changes in triglyceride, total cholesterol, LDL-C, and HDL-C did not differ between the groups after treatment (*p* > 0.05) (Table 2).

Lactic acidosis and vitamin B12 deficiency was not observed during treatment. Gastrointestinal discomfort (17.2%) was observed in the metformin-insulin group. Hypoglycemic events did not differ between the groups (7.1 and 7.6%, *p* > 0.05).

The decrease in metabolic syndrome prevalence was not correlated with a reduction in insulin dose requirement (*r* = 0.45, *p* = 0.23). The decrease in insulin dose requirement was positively correlated with the reduction in fasting glucose (*r* = 0.487, *p* < 0.001) and postprandial glucose (*r* = 0.774, *p* < 0.001).

## Discussion

This retrospective study suggests that metformin decreased glucose concentrations, lowered metabolic syndrome prevalence, as well as insulin dose requirement more than insulin therapy alone after 1 year of treatment. These results were independent of blood lipid improvement or weight loss, although on average weight remained decreased with metformin and insulin therapy, whereas the average weight increased with insulin therapy alone.

Studies of adolescents [18], pediatrics [22], adults [4], and overweight adults [19–21] with type 1 diabetes have been shown the addition of metformin to insulin therapy to reduce insulin dose requirement. Metformin was suggested to reduce insulin dose requirement through its insulin-sparing effect [4, 15, 25]. The present study suggests that metformin decreased average glucose concentrations and insulin dose requirement, as well as lowered metabolic syndrome prevalence more than with insulin therapy alone after 1 year of treatment. Metformin decreased HbA1c values more than insulin alone, but not significantly. These results were independent of blood lipid improvement or weight loss. We suggest that metformin likely improved glycemic control more than with insulin alone and this also contributed to metabolic syndrome reductions. Metformin has been shown to have an insulin-sensitizing effect on glycemic control in type 1 diabetes [17, 20]. Nevertheless, metformin has been shown to reduce insulin dose requirement without improving glycemic control such as with glucose concentration, as well as HbA1c in type 1 diabetes [4, 20, 21]. Jacobsen et al. reported that metformin reduced the insulin dose requirement without improving glycemic control or weight loss after 6 months’ treatment in overweight adults with type 1 diabetic [21]. Lund et al. suggested that metformin achieved permanent inadequate glycemic control with reducing insulin dose requirement and improved body weight during 12 months’ treatment [4]. Urakami et al. observed that metformin reduced insulin dose requirement, improved glycemic control, and reduced body weight after 12 months’ treatment in overweight young adults with type 1 diabetes [25]. Moon et al. reported that metformin improved glycemic control and insulin sensitivity without weight gain after 3 months’ therapy [26]. Metformin improved diabetic control with reduced insulin dose requirement without weight loss in overweight adults with C-peptide-negative type 1 diabetes during 4 months’ therapy [19]. Metformin improved poor glycemic control as well as insulin resistance in adolescents with type 1 diabetes who were on high-dose insulin therapy [17, 18].

Previous studies reported that metformin improved the blood lipid profile [26–28]. Burchardt et al. reported that metformin increased insulin sensitivity in peripheral tissues and reduced LDL-C concentrations with improved glycemic regulation, as well as weight loss, in overweight adults [27]. The present study suggests that metformin reduced glucose concentration and decreased insulin dose requirement without blood lipid improvement and weight loss. On average weight remained decreased on metformin and insulin therapy, whereas the average weight increased in the insulin alone group. There was a decrease in relatively normal lipid values in the metformin-insulin group compared with the insulin alone group; however, these results were not significant. There was a greater increase in systolic and diastolic blood pressure in the insulin alone group compared with the metformin-insulin group. These results might contribute to improved metabolic syndrome status with metformin therapy.

Previous studies have not systematically tested metformin in patients with metabolic syndrome with T1DM. Metformin likely reduced metabolic syndrome percentage in present study. Insulin resistance accompanied by type 1 diabetes causes the development of double diabetes and poorer glycemic control [5, 6, 9, 29]. Metformin reduces hepatic glucose production, stimulates glucose uptake in muscle, as well as improves blood flow for nutrient use. Metformin increases insulin sensitivity, reduces insulin resistance, improves insulin action, and increases peripheral glucose uptake in type 1 diabetes [18, 20, 21, 26]. Patients with C peptide-negative diabetes were included in this study in order to ignore metformin effects on beta cell function; metformin correlates with insulin resistance and beta-cell function. Hypoglycemic events [4, 20–22], lactic acidosis or vitamin B12 deficiency [26] were not observed in our metformin therapy group. However, this was a small and retrospective study, the study was not randomized or placebo-controlled and diet was not standardized prior to testing. All of which are limitations to our study.

## Conclusions

The present study suggests that metformin decreased glucose concentrations, lowered metabolic syndrome prevalence, as well as insulin dose requirement, more than insulin alone. These effects were independent of blood lipid improvement or weight loss, although on average weight remained decreased with metformin and insulin therapy, whereas the average weight increased with insulin therapy alone. Larger placebo-controlled studies are needed to determine the long-term effects of metformin-adjunctive therapy on poorly controlled type 1 diabetes.

## Abbreviations

BMI: Body mass index
BP: Blood pressure
DM: Diabetes mellitus
FBP: Fasting plasma glucose
HbA_1c_: Hemoglobin A_1c_
HDL-C: High-density lipoprotein-cholesterol
LDL-C: Low-density lipoprotein-cholesterol
PPG: Postprandial plasma glucose
TG: Triglyceride
WC: Waist-circumference
